# Acute Fulminant Necrotizing Amoebic Colitis Leading to Intestinal Perforation and Peritonitis

**Published:** 2015-01-01

**Authors:** Prince Raj, Yogesh Kumar Sarin

**Affiliations:** Department of Pediatric Surgery, Maulana Azad Medical College, and associated Lok Nayak Hospital, New Delhi, India.

**Dear Sir,**

Invasive intestinal amoebiasis presenting as cecal perforation is a rare entity in children. It is associated with high mortality and dismal outcome.[1] We hereby highlight a case of 4-year old child with amoebic colitis causing cecal perforation and peritonitis.

A 4-year old boy presented with acute abdomen for 2 days. There was a history of recurrent abdominal pain for 2 weeks. After initial resuscitation, abdominal radiograph was done, which showed multiple air-fluid levels. Ultrasonography showed dilated bowel loops with thickened wall. Blood investigations revealed anemia and markedly raised C-reactive protein. After optimization, laparotomy was performed which revealed gangrenous ileo-cecal region and perforations in cecum and terminal ileum, with foul smelling contents. Ileostomy and ascending colostomy was performed after resecting ileo-cecal region. In the post-operative period, foul-smelling contents kept coming out through the distal stoma for a week. Colonoscopy was done through the distal stoma, which revealed edematous and ulcerated ascending colon. Biopsy showed the presence of flask-shaped ulcers suggestive of amoebic colitis (Fig.1). Metronidazole and diloxanide furoate were started and the child gradually improved. He was discharged after 11 days of admission and a month later distal colostogram was done, following which stoma was closed.

**Figure:**
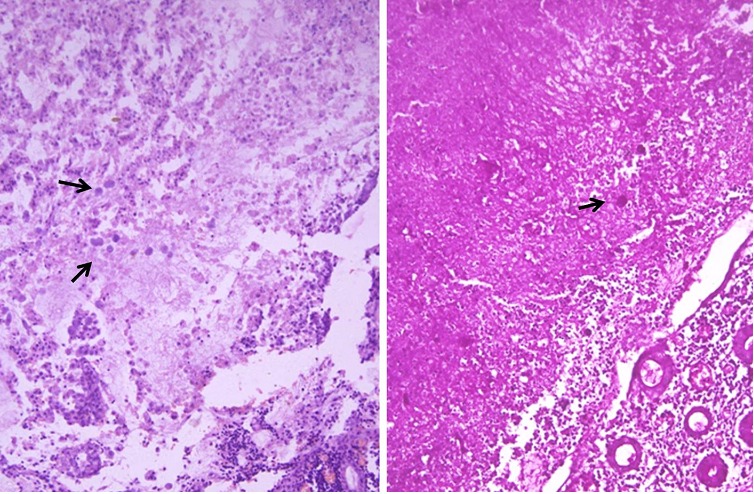
Figure 1:Multiple amoebic trophozoites in the necrotic area surrounded by acute inflammation.(Haematoxylin/Eosin x 400). Amoebic trophozoites highlighted magenta pink (PAS stain x400)

Intestinal amoebiasis specifically affects the colon with predilection for right side and 6-11% of patients with invasive form may require surgical intervention.[1] Most of the times, it is asymptomatic. On the other end of the spectrum acute fulminant colitis with perforation, the most dreaded and feared complication associated with very high mortality rate, may result.[1-5] Management is a bit challenging, as there is low threshold of suspicion for amoebiasis as a cause of perforation, and the diagnosis is made intra-operatively or after the biopsy report. Intra-operatively, the diseased colon is extensively friable and disintegrates with simple manipulation, as happened in our case.[3,4] Eggleston et al and Lubyuski et al advocated diversion and drainage as the main procedure, with resection reserved for gangrenous bowel; mortality associated with resection in these two studies were 71% and 83% respectively.[2,5] The best management for children with amoebic colitis perforation would be the resection of gangrenous colon with stoma formation, as this will not only remove the diseased segment, but also remove the septic foci and prevent further fecal contamination. Once the patient recovers, intestinal continuity may be restored later on after performing distal colostogram to confirm distal patency. Primary repair should always be discouraged as the chances of anastomotic dehiscence are very high due to inflamed and friable bowel.

## Footnotes

**Source of Support:** Nil

**Conflict of Interest:** None declared

